# Association of birth defects during the perinatal period with child mortality under 5 years

**DOI:** 10.3389/fped.2025.1485176

**Published:** 2025-07-31

**Authors:** Donghua Xie, Jianhui Wei, Lili Xiong, Huiyuan Zhu, Xianglian Peng, Bo Li, Kehan Zou

**Affiliations:** ^1^ Department of Health Management, Maternal and Child Health Hospital of Hunan Province, Changsha, China; ^2^ NHC Key Laboratory of Birth Defects for Research and Prevention, Hunan Provincial Maternal and Child Health Care Hospital, Changsha, Hunan, China; ^3^ Xiangya School of Public Health, Central South University, Changsha, China; ^4^ School of Public Health, University of South China, Hengyang, China; ^5^ Clinical Medical Research Center of Maternal Neonatal Diseases of Hunan Province, Maternal and Child Health Hospital of Hunan Province, Changsha, Hunan, China

**Keywords:** birth defects, mortality rate under 5 years, cause of death, hazard ratio, confidence intervals, person-years

## Abstract

**Objective:**

To calculate the impact of birth defects (BDs) diagnosed during the perinatal period on the mortality of children under 5 years of age.

**Methods:**

This was a retrospective cohort analysis. From the monitoring system, we collected all hospital delivery, BD monitoring, and death information for children under 5 years in Hunan Province from 2017–2022. These data were linked by ID number. Mortality rates and Cox proportional hazards models were used to compute hazard ratios (HRs) and 95% confidence intervals (CIs) for the impact of BDs on mortality in children under 5 years of age.

**Results:**

Among 3,807,340 live-born children, 29,879 (0.8%) had at least one type of BDs during the perinatal period, with a total of 12,215,033 person-years of follow-up. The mortality rate of the BDs group was 14.5% (95% CI: 13.7–15.3) per 1,000 person-years, which was 11.6 times (HR = 11.6, 95% CI: 10.5–12.8) greater than that of the nondefect group. The mortality rate per 1,000 person-years of girls with BDs was higher than that of boys (15.4% vs. 13.5%). For the BDs group, congenital anomalies (CAs) were the most common cause of death (57.2%). Compared with children without BDs, those with BDs had elevated mortality risks for CAs (HR, 58.1; 95% CI, 42.7–79.0), digestive (HR = 16.5, 95% CI: 6.1–45.0) and respiratory system malformations (HR, 11.9; 95% CI, 7.9–17.8), and cancer (HR = 11.1 95% CI: 4.7–26.2).

**Conclusions:**

This study revealed that BDs were strongly associated with mortality under 5 years of age, especially in the first 28 days, for muscular, chromosomal, genetic, and nervous system abnormalities.

## Introduction

In the past 20 years, progress has been made in child health, with the global under-5 mortality rate (U_5_MR) decreasing by nearly 60% (1). Based on estimates published by the UN Interagency Group on Child Mortality Estimation (UNIGME), China's U_5_MR declined from 53.8–7.1 per 1,000 live births, with an average annual rate of reduction (ARR) of 6.5% from 1990–2021 (2, 3, 4). However, the relative contribution of congenital anomalies (CAs) to child mortality is increasing globally and has therefore been highlighted as an emerging priority to be addressed by the UN Sustainable Development Goals in the post-2015 child health agenda (3).

**Figure 1:**
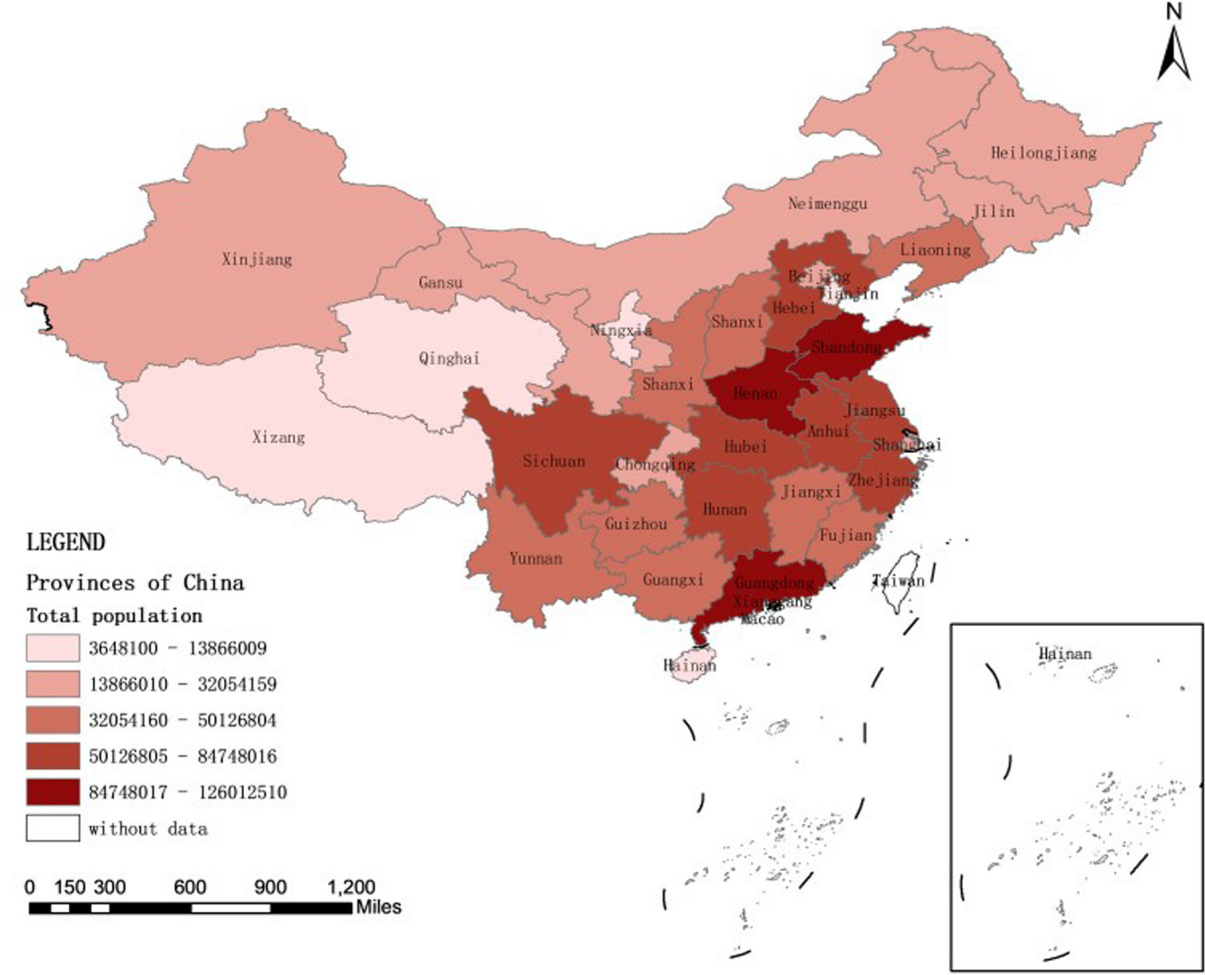
The map of population distribution by province of the 7th census of China.

There have been several studies on the association between birth defects (BDs) and child mortality, especially during infancy (5, 6). According to research, CAs were the main cause of death among children under 14 years of age (21.8%) (7) and accounted for 20.6% of infant deaths in 2017 in the USA (8). However, these data cannot indicate the probability that live-born children with BDs die at different ages than healthy children do, which is important for clinician counseling. A quantitative summary of population-based studies of survival beyond infancy for children with specific CAs is lacking, except for congenital heart disease (CHD) (9), neural tube defects (NTDs) (10, 11) and trisomies (12). To answer these questions, there is a need to establish population-wide cohorts with BDs and normal groups and then follow-up the groups for at least several years. In addition, The systematic analysis revealed that great inequity exists in child mortality across regions and in urban vs. rural areas in China (13). Therefore, it is necessary to analyze the association between BDs during perinatal and U_5_MR in Hunan Province, which is a relatively densely populated central province, representative of the average level of Chinese economy (Figure 1).

At present, most countries have relatively mature monitoring systems for BDs and deaths of children under 5 years of age (10, 14); however, they are essentially independent rather than population-based monitoring systems, so it is difficult to establish a complete database for children from birth to death. Hunan Province, located in southern China, has had a birth information system covering all midwifery institutions in the province since 2017. All midwifery institutions are monitored for BDs, and all deaths of children under 5 years of age are monitored in Hunan Province. Therefore, based on birth information, the BD monitoring system, and the database recording deaths of children under 5 years of age, we can evaluate the impact of all BD types on the U_5_MR in the population.

## Methods

### Study design and population

This was a retrospective cohort analysis. The maternal and child health direct reporting information system of Hunan Province was instituted in 2017 by the Health Commission of Hunan Province (HCHP). From the three independent databases of this system, we collected all hospital delivery, BD monitoring, and death information of children under 5 years in Hunan Province from 2017–2022. The hospital delivery information was linked to the BD monitoring and death information of children under 5 years of age by ID number, which was unique for every person. When a pregnant mother has multiple deliveries or gives birth to multiple babies in one delivery, the birth time of the children should be matched precisely, down to the minute. We did not include stillbirths or terminations of pregnancy (TOPs). The sample was a population study in Hunan Province, which is located in southern China. This study was approved by the Ethics Committee of Maternal and Child Care, Hospital of Hunan Province (2021-S056). All personal information was removed after database integration but before analysis.

### Hospital deliveries

According to the requirements of the HCHP, every hospital needs to input delivery information for every mother within 3 days after delivery. The delivery information includes 2 parts: maternal information and newborn information. The maternal information includes name, age, ID number, location, nationality, education level, gestational week, delivery date, delivery model, and pregnancy outcome. Newborn information includes weight, height, and Apgar scores.

### Birth defects

Infants with BDs were identified in obstetrics or neonatal departments according to the Maternal and Child Health Monitoring Scheme in China (MCHMSC), developed by the National Health System. The monitoring time was the perinatal period (from 28 weeks to 7 days after birth) in China. The case cards were completed by gynecological, pediatric, or neonatal doctors. Every case card for an infant with BDs included 5 parts: maternal information (such as the mother's name, ID number, residence, and education level), infant birth information, BD diagnoses, maternal illnesses and drug use during pregnancy, and a detailed family disease history. The case cards were reported quarterly, both on paper and online, and were audited by the maternal and child health hospitals and health administrative departments, respectively, step by step. The diagnosis of BDs was based on the MCHMSC. Birth defect surveillance encompasses structural and functional malformations. The principle for case inclusion is that the defect has a high incidence rate, or poses a certain degree of harm, or incurs a significant disease burden. Non-specific symptoms, signs, imaging features, auxiliary examination results, and suspected diagnoses are not included as confirmed diseases. Congenital infections that do not cause physical structural malformations, metabolic function defects, or intellectual disabilities in newborns/fetuses (such as syphilis, hepatitis B, etc.) are not reported as birth defect cases.Simple patent foramen ovale and patent ductus arteriosus with dimension ≤5 mm were not included.

About the most common type of congenital heart disease,during the period from 2017–2022, the diagnosis of congenital heart disease in Hunan Province mainly followed the following process: NT screening in the early pregnancy, fetal cardiac ultrasound for major anomaly screening in the second trimester, ultrasound re-examination of heart malformations in the third trimester, and after birth, based on whether there were any abnormal clinical symptoms, echocardiography diagnosis was conducted.

We coded BDs with the International Statistical Classification of Diseases and Related Health Problems, 10th Revision (ICD-10). We categorized defects by organ system: congenital malformations of the nervous system (Q00‒Q05); cleft lip and palate (Q35‒Q37); congenital malformations of the eyes, ears, face and neck (Q11‒Q12‒Q16‒Q17), circulatory system (Q20‒Q26), digestive system (Q39‒Q42), urinary system (Q53‒Q54‒Q56‒Q64), skeletal system (Q66‒Q72), and muscular system (Q79); and chromosomal and genetic abnormalities (Q90‒Q99). We considered children who had more than 1 type of BD as having multiple BDs. The comparison group included children without BDs during the perinatal period.

### Causes of death

In accordance with China's basic public health management rules, town and township hospitals and community healthcare centers need to provide health management for 0- to 6-year-old children. When children die, these facilities need to collect information from the hospitals in which the deaths occurred and investigate the deaths by contacting the children's parents. Then, they report the death case cards both on paper and online. Death case cards include parent names and ID numbers, child sex, birth date, birth weight, gestational age, location, death age, death data, death location, primary cause of death, and diagnosis basis.

The cause of death is recorded on the discharge summary with the ICD-10 codes. We categorized deaths by cause: infection (A00–B99); cancer (C00–D48); CAs (Q00–Q99); nervous (G00–G99), digestive (K00–K93), and respiratory system abnormalities (J00–J99); injuries and external causes (IECs) (S00–T98 and V01–Y98); perinatal causes (P00–P96); blood abnormalities (D50–D89); endocrine, nutritional, and metabolic causes (E00–E90); circulatory (I00–I99), musculoskeletal (M00–M99), and genitourinary abnormalities (N00–N99); and shock and ill-defined conditions (R00–R99). In our study, because there were fewer deaths from blood, endocrine, nutritional, metabolic, circulatory, musculoskeletal, and genitourinary abnormalities; shock; and ill-defined conditions, we classified these deaths as others. In addition, considering the BD monitoring time, CAs were divided into CAs/BDs diagnosed during the perinatal period and CAs diagnosed more than 7 days after birth.

### Information data quality control

Every month, the maternity institutions verify the accuracy, timeliness and consistency of the information entered into the case cards for inpatient deliveries and birth defect monitoring through inpatient medical records. Every month, the township health centers or community health service centers verify the accuracy, timeliness and consistency of the reported cases of deaths of children under 5 years old through death clues from comprehensive medical institutions and death clues reported by village doctors. Every quarter, the district and county maternal and child health hospitals verify the birth and death information reported by maternity institutions and township/community institutions through the public security system and the local electronic case system. At the same time, they conduct on-site verification of the quality of the reported information on birth defects cases at each maternity institution. The municipal and provincial maternal and child health hospitals verify the birth and death information and birth defect monitoring information of children under 5 years old through the provincial electronic case home page and the provincial disease control system every half year. For regions with more problems in each city and state, on-site quality control will be conducted. Throughout the year, the provincial level conducts full coverage of on-site quality control at the municipal and state levels, and the municipal and state levels conduct full coverage at the district and county levels.

### Statistical analysis

Due to the inconsistent length of the observation period, we used the mortality rate (MR) per 1,000 person-years(number of deaths/the total number of years that all the children lived until 2022.12.31 *1000) MRs and Cox proportional hazards models were used to compute hazard ratios (HRs) and 95% confidence intervals (CIs) separately for the associations between BDs and mortality in boys, girls and the total population. Cox proportional hazards models were used for each type of BD diagnosed during the perinatal period, and both all-cause and cause-specific mortalities were examined. The time scale was measured as the number of days between the date of birth and the date of death or the study end date of December 31, 2022. Some of the newborns born in 2017 were over 5 years old, and the survival times were all up to 5 years of age. Constituent ratios were used to describe the distribution of the causes of death. We adjusted the models for maternal age, multiple births, socioeconomic deprivation, education level, preterm birth (<37 and ≥37 gestational weeks), and birth weight. According to the time limits for neonates, infants and young children, to illustrate the mortality risk for different BDs at different times, we stratified the analysis by age at death (≤28 days, 29–364 days, 1–2 years, and 3–5 years). We performed the analysis with R 4.0.2 and assessed statistical significance with 95% CIs.

## Results

Among 3,807,340 live-born infants born from 2017 to 2022, 29,879 (0.8%) had at least one type of BDs during the perinatal period. All live-born infants were followed until 5 years of age or December 31, 2022, with a total of 12,215,033 person-years of follow-up. A total of 16,275 (0.4%) children died in the first 5 years of life, including 1,354 (4.5%) in the BD group. The mortality rate of the BDs group was 14.5% (95% CI: 13.7–15.3) per 1,000 person-years, which was 11.6 times greater than that of the nondefective group (HR = 11.6, 95% CI: 10.5–12.8). The mortality rate per 1000 person-years of girls with BDs was higher than that of boys (15.4% vs. 13.5%). The U_5_MR of muscular abnormalities was the highest [MR = 112.5 (95% CI: 86.6–144.7) per 1,000 person-years] in children with BDs compared with those without BDs [HR = 57.4 (95% CI: 42.9–76.8)], followed by chromosomal and genetic abnormalities [MR = 93.4 (95% CI: 72.4–119.5) per 1,000 person-years, HR = 40.5 (95% CI: 30.2–54.2)] and nervous system abnormalities [MR = 66.1 (95%CI: 52.7–82.6) per 1,000 person-years, HR = 46.83 (95% CI: 36.1–60.7)]. Children with multiple BDs had a greater risk of death than those with isolated BDs did [HR = 32.8 (28.4–37.9) vs. 9.35 (8.4–10.4)] (Table 1).

**Table 1:** Associations between BDs and the U5MR.

Type of BDs	No. of deaths/No. of children with BDs (%)			Mortality rate per 1,000 person-years (95% CI)			HR (95% CI)		
	Boys	Girls	Total	Boys	Girls	Total	Boys	Girls	Total
Any	768/17,990 (4.3)	566/11,827 (4.8)	1,354/29,879 (4.5)	13.5 (12.6–14.5)	15.4 (14.2–16.7)	14.5 (13.7–15.3)	9.8 (8.6–11.2)	14.0 (12.0–16.4)	11.58 (10.48–12.80)
Nervous System	45/222 (20.3)	29/155 (18.7)	75/378 (19.8)	69.1 (51.3–91.9)	60.1 (41.4–86.3)	66.1 (52.7–82.6)	45.6 (32.9–63.3)	49.0 (32.0–75.0)	46.83 (36.12–60.72)
Cleft lip and palate	93/1,162 (8.0)	72/983 (7.3)	166/2,148 (7.7)	26.3 (21.4–32.3)	24.3 (19.2–30.7)	25.5 (21.9–29.7)	14.3 (11.2–18.3)	214 (16.3–28.1)	17.27 (14.38–20.74)
Eyes, ears, face and neck	71/2,086 (3.4)	52/1,387 (3.7)	124/3,474 (3.6)	10.5 (8.3–13.3)	11.4 (8.6–15.0)	10.9 (9.1–13.0)	9.8 (7.5–12.7)	13.7 (9.8–18.3)	11.18 (9.14–13.68)
Digestive	85/641 (13.3)	61/260 (23.5)	150/906 (16.6)	47.9 (38.7–59.2)	100.7 (78.5–128.1)	62.9 (53.6–73.6)	30.1 (23.4–38.7)	58.7 (428–80.7)	39.62 (32.67–48.06)
Urinary	80/1,690 (4.7)	26/409 (6.4)	107/2,106 (5.1)	16.5 (13.3–20.6)	23.6 (15.8–34.9)	17.9 (14.8–21.7)	7.3 (5.6–9.6)	11.7 (7.2–18.9)	8.33 (60.61–10.50)
Skeletal	116/6,714 (1.7)	96/4,134 (2.3)	215/10,852 (2.0)	5.3 (4.4–6.4)	7.2 (5.9–8.8)	6.1 (5.3–7.0)	4.2 (3.3–5.2)	6.8 (5.3–8.8)	5.19 (4.41–6.12)
Muscular	25/131 (19.1)	30/76 (39.5)	55/207 (26.6)	73.1 (48.8–107.4)	204.4 (144.0–280.1)	112.5 (86.6–144.7)	37.2 (24.6–56.4)	102.81 (67.4–156.9)	57.37 (42.85–76.81)
CHD	215/3,002 (7.2)	162/2,689 (6.0)	378/5,695 (6.6)	25.5 (22.8–29.8)	21.4 (18.3–25.0)	23.6 (21.3–26.1)	14.33 (11.8217.4)	16.3 (12.9–20.4)	15.13 (13.07–17.52)
Chromosomal and genetic	33/147 (22.4)	26/136 (19.1)	59/284 (20.8)	98.0 (69.3–136.0)	89.3 (60.2–129.6)	93.4 (72.4–119.5)	38.9 (26.4–57.5)	44.0 (28.2–68.7)	40.45 (30.17–54.24)
Others	251/3,879 (6.5)	191/2,601 (7.3)	461/6,534 (7.1)	19.4 (17.1–22.0)	23.2 (20.1–26.7)	21.6 (19.7–23.7)	15.4 (13.0–18.4)	21.1 (17.3–25.8)	18.28 (16.06–20.81)
Isolated	571/16,443 (3.5)	428/10,906 (3.9)	1,010/27,396 (3.7)	10.9 (10.1–11.8)	12.5 (11.4–13.8)	11.7 (11.0–12.4)	7.9 (6.7–9.1)	11.5 (9.7–13.5)	9.35 (8.41–10.40)
Multiple	197/1,547 (12.7)	138/921 (15.0)	344/2,483 (13.9)	44.1 (42.7–45.6)	54.9 (46.5–64.7)	49.2 (44.3–54.6)	26.4 (21.8–32.0)	43.7 (34.8–54.8)	32.84 (28.42–37.94)
None within 7 days	8,515/19,66,017 (0.4)	6,407/17,43,449 (0.4)	14,921/37,77,461 (0.4)	1.3 (1.3–1.3)	1.1 (1.1–1.1)	1.2 (1.2–1.2)	1 (Reference)	1 (Reference)	1 (Reference)

HR for any BD vs. no defect, adjusted for maternal age, multiple births, socioeconomic deprivation, education level, preterm birth, and birth weight.

CHD, congenital heart disease.

Among the 1,354 children with BDs who died, CAs were the most common cause of death (57.2%), followed by perinatal diseases (18.3%). Among death with CAs, the most common category was muscular abnormalities (89.1%), digestive abnormalities (75.3%), and congenital heart disease (CHD).Among the 14,921 children without BDs who died, perinatal disease was the most common cause of death (29.0%), followed by IECs (28.0%) and BDs (13.2%). Overall, among the total live births, BDs accounted for 4.8% of the deaths (774/16,275), and CAs, regardless of the time of diagnosis, accounted for 16.9% of the deaths (2,747/16,275) (Table 2).

**Table 2:** Distribution of causes of death of the 1,354 children with BDs and 14,921 children without BDs.

Type of BDs	No. of deaths by cause (%)									
	CA	Nervous system abnormalities	Respiratory abnormalities	Digestive abnormalities	Infection	Cancer	IECs	Perinatal causes	Others	Total
Any	774 (57.2)	11 (0.8)	114 (8.4)	25 (1.8)	19 (1.4)	27 (2.0)	77 (5.7)	248 (18.3)	59 (4.4)	1,354 (100.0)
Nervous system	46 (61.3)	3 (4.0)	4 (5.3)	0 (0.0)	1 (1.3)	3 (4.0)	3 (4.0)	13 (17.3)	2 (2.7)	75 (100.0)
Cleft lip and palate	102 (61.4)	0 (0.0)	13 (7.8)	3 (1.8)	2 (1.2)	0 (0.0)	16 (9.6)	26 (15.7)	4 (2.4)	166 (100.0)
Eyes, ears, face and neck	62 (50.0)	0 (0.0)	9 (7.3)	2 (1.6)	0 (0.0)	2 (1.6)	16 (12.9)	28 (22.6)	5 (4.0)	124 (100.0)
Digestive	113 (75.3)	0 (0.0)	5 (3.3)	4 (2.7)	2 (1.3)	1 (0.7)	2 (1.3)	23 (15.3)	0 (0.0)	150 (100.0)
Urinary	53 (49.5)	1 (0.9)	4 (3.7)	2 (1.9)	2 (1.9)	2 (1.9)	5 (4.7)	30 (28.0)	8 (7.5)	107 (100.0)
Skeletal	89 (41.4)	1 (0.5)	29 (13.5)	7 (3.3)	4 (1.9)	3 (1.4)	24 (11.2)	48 (22.3)	10 (4.7)	215 (100.0)
Muscular	49 (89.1)	0 (0.0)	2 (3.6)	0 (0.0)	0 (0.0)	1 (1.8)	0 (0.0)	3 (5.5)	0 (0.0)	55 (100.0)
CHD	272 (72.0)	3 (0.8)	26 (6.9)	3 (0.8)	6 (1.6)	2 (0.5)	7 (1.9)	50 (13.2)	9 (2.4)	378 (100.0)
Chromosomal and genetic	35 (59.3)	1 (1.7)	7 (11.9)	0 (0.0)	0 (0.0)	1 (1.7)	3 (5.1)	10 (16.9)	2 (3.4)	59 (100.0)
Others	247 (53.6)	5 (1.1)	38 (8.2)	11 (2.4)	6 (1.3)	14 (3.0)	14 (3.0)	95 (20.6)	31 (6.7)	461 (100.0)
None within 7 days	1,973 (13.2)^a^	413 (2.8)	1,798 (12.1)	372 (2.5)	444 (3.0)	519 (3.5)	4,171 (28.0)	4,327 (29.0)	904 (6.1)	14,921 (100.0)

^a^ CAs were diagnosed during the perinatal period.

BDs were associated with an increased risk of death from most causes (HR = 11.6, 95% CI: 10.5–12.8) (Table 3). Compared with children without BDs, those with BDs had elevated risks of mortality from CAs (HR, 58.1; 95% CI, 42.7–79.0), digestive abnormalities (HR = 16.5, 95% CI: 6.1–45.0), respiratory abnormalities (HR, 11.9; 95% CI, 7.9–17.8), and cancer (HR = 11.1 95% CI: 4.7–26.2) (Table 3).

**Table 3:** Association between BDs and cause-specific characteristics of the U_5_MR.

Cause of death	No. of deaths (% of children who died)		Hazard ratio (95% CI)^b^
	Children with BDs	Children without BDs	
All causes	1,354 (4.5)	14,921 (0.4)	11.6 (10.5–12.8)
CA	774 (2.6)	1,973 (0.1)^a^	58.1 (42.7–79.0)
Central nervous system	11 (0.0)	413 (0.0)	6.7 (1.9–23.4)
Respiratory	114 (0.4)	1,798 (0.1)	11.9 (8.0–17.8)
Digestive	25 (0.1)	372 (0.0)	16.5 (6.1–45.01)
Infection	19 (0.1)	444 (0.0)	3.5 (1.6–7.5)
Cancer	27 (0.1)	519 (0.0)	11.1 (4.7–26.2)
IECs	77 (0.3)	4,171 (0.1)	3.9 (2.7–5.5)
Perinatal disease	248 (0.8)	4,327 (0.1)	5.6 (4.4–7.2)
Other reasons and acatalepsy	59 (0.2)	904(0.0)	10.0(5.9–17.2)

^a^ CAs were diagnosed during the perinatal period.

^b^ HR for any BD vs. no defect, adjusted for maternal age, sex, multiple birth, socioeconomic deprivation, education level, preterm birth, and birth weight.

Children with BDs had a high risk of mortality at all ages, especially in the first year after birth. Compared with children without BDs during the perinatal period, those with any BDs had 19.1 times (95% CI: 16.0–22.8) the risk of mortality in the first 28 days after birth, 11.87 times (95% CI: 9.3–15.1) the risk between 29 and 364 days, 3.09 times (95% CI: 2.2–4.4) the risk between 1 and 2 years, and 2.98 times (95% CI: 1.9–4.7) the risk between 3 and 5 years. These trends were observed for most types of BDs (Table 4).

**Table 4:** Association between BDs and mortality according to age at death.

Type of BDs	Hazard ratio (95% CI)^a^			
	≤28 days	29–364 days	1–2 years	3–5 years
Any	19.1 (16.0–22.8)	11.9 (9.3–15.1)	3.1 (2.2–4.4)	3.0 (1.9–4.7)
Central nervous system	438 (31.8–60.2)	26.8 (13.6–52.7)	28.3 (11.4–70.8)	19.5 (2.6–146.2)
Cleft lip and palate	18.8 (14.8–23.9)	14.0 (9.9–19.7)	2.3 (0.7–7.5)	2.8 (0.4–21.0)
Eyes, ears, face and neck	16.7 (13.0–21.5)	6.4 (4.1–9.9)	3.1 (1.7–7.2)	1.8 (0.2–12.9)
Digestive	48.8 (38.5–61.9)	16.2 (10.1–26.1)	2.1 (0.3–15.4)	*n* = 4/NA*
Urinary	6.0 (4.3–8.3)	5.7 (4.4–7.3)	4.7 (1.9–11.3)	*n* = 0/NA
Skeletal	6.3 (5.1–7.8)	3.4 (2.5–4.7)	2.3 (1.3–4.0)	1.10 (0.3–4.7)
Muscular	71.0 (50.5–100.3)	22.2 (10.2–48.2)	*n* = 1 NA*	*n* = 0/NA
CHD	16.7 (13.7–20.4)	13.6 (10.4–17.6)	3.5 (1.8–7.0)	1.0 (0.9–1.0)
Chromosomal and genetic	34.1 (23.0–50.1)	39.0 (23.3–65.5)	29.6 (10.5–831)	37.9 (5.0–287.1)
Others	27.4 (23.1–32.5)	11.0 (8.5–14.3)	2.5 (1.2–5.0)	0.9 (0.1–6.3)
None within 7 days	1 (Reference)	1 (Reference)	1 (Reference)	1 (Reference)

*NA, not applicable.

^a^ HR for any BD vs. no defect, adjusted for maternal age, sex, multiple birth, socioeconomic deprivation, education level, preterm birth, and birth weight.

## Discussion

This was a retrospective cohort study with 3,807,340 live-born infants and a total of 12,215,033 person-years of follow-up. The results revealed that the mortality rate of the BD group was 14.5% (95% CI: 13.7–15.3) per 1,000 person-years, which was 11.6 times greater than that of the nondefective group. Until now, except for the U_1_MR and U_14_MR, there have been few reports about the HRs of BDs for the U_5_MR. A population study in Texas showed that the 10-year survival rate of infants with major birth defects was 93.9 (15). A study published in the Journal of Pediatrics showed the infant survival probability of 21 types of BDs, such as 91.9% for spina bifida without anencephaly (16). A study by Alessio Coi revealed the survival rate of children born with rare structural CAs in western Europe, which exhibited considerable variation between individual anomalies (17). A study by Marie-Laure Sattolo revealed that children with BDs had a high risk of mortality between birth and the age of 14 years. Compared with the normal group, boys with BDs had a 4.69-fold greater risk of death, whereas girls had a 5.66-fold greater risk (7). Due to differences in sex chromosomes between female and male infants, their epigenetic and biological susceptibilities vary (18), ultimately leading to differences in mortality rates from different types of birth defects. In addition, BD mortality is different in different countries. India contributed 21% of BDs in the early neonatal period worldwide, with 20% (11,864 out of 58,040) of deaths in the late neonatal period, 14% in the postneonatal period and 8% in the 1- to 4-year-old age group (19).

The U_5_MR of muscular abnormalities was the highest (HR = 57.4), followed by chromosomal and genetic abnormalities (HR = 40.5) and nervous system abnormalities (HR = 46.8). The survival rates from early pregnancy to 28 days and 1 year were similar to those reported in another study, which revealed that lung hypoplasia, congenital diaphragmatic hernia and gastroschisis were the most common causes of infant death (5, 20). Children with prune belly syndrome had consistently low survival across all age points, declining from 76.4% at 1 week to 67.0% at 1 year and further to 57.4% at 10 years of age (17). Congenital diaphragmatic hernia, gastroschisis, and prune belly syndrome are muscular abnormalities that can affect the circulatory, hemodynamic, and digestive systems immediately after birth. In addition, it is acknowledged that there are very few long-term survivors of nonmosaic trisomy 21, trisomy 13 and trisomy 18 (21). The chances of survival to 28 days and 1 year are 6.2% and 0.2% for trisomy 18 and 10.3% and 3.0% for trisomy 13, respectively (21).

Among the 1,354 children with BDs who died, CAs were the most common cause of death (57.2%), followed by perinatal diseases (18.3%). Among the total live births, BDs accounted for 4.8% of the deaths, and CAs, regardless of the time of diagnosis, accounted for 16.9%. A meta-analysis by Chunhua He revealed that the leading cause of death for the U_5_MR in 2015 in China was CAs (19.7%) (2), which is similar to those reported in high-income countries, such as the USA (22). A systematic review in 2019 by Jamie Perin revealed that intrapartum-related events accounted for 11.6% of the U_5_MR based on 5.30 million deaths in children under 5 years of age (23), which was lower than that reported in our study. The reason for this difference, other than differences in the management of BDs and child heath, may be the use of different BD monitoring systems involving different monitoring periods. In China, the period of BD monitoring is from 28 weeks of pregnancy to 7 days after birth. The BD monitoring periods are mainly from 20 weeks of pregnancy to 6 years after birth in Australia and from 20 weeks of pregnancy to 1 year after birth in Finland. In addition, TOPs are not permitted in some countries, such as Malta and nations in South America, which increased BD mortality. Therefore, when we compare BD mortality rates or the proportions of causes of death, we should consider monitoring rules and TOP policies.

With respect to the distribution of causes of death, perinatal diseases (18.3% and 29.0% of infants with and without BDs, respectively) and injuries (5.7% and 28.0% of infants with and without BDs, respectively) were the most common causes of death, which were similar to the findings of other studies. After BDs, the most frequently listed causes of neonatal death were preterm birth/low birth weight (10%), circulatory system diseases (8%), and sepsis (5%), which are perinatal diseases (5). A meta-analysis by Chunhua He revealed that injuries became the leading cause of death, contributing to 41.1% of deaths in children aged 12–23 months and 53.8% of deaths in children aged 24–59 months (2). Therefore, in addition to BDs, child survival policies and programs should clearly concentrate on preventing and treating injuries.

In conclusion, this study revealed that children with BDs during the perinatal period have a serious impact on the U_5_MR, especially for the first 28 days, which was similar to the findings of other studies. A study by Dhammasagar Ujagare revealed that BD mortality was highest in the early neonatal period in India (19). Additionally, the proportion of neonatal deaths among children who died before the age of 5 years in China was more than 50% (2). Therefore, prevention and treatment of neonatal diseases are key to reduce the U_5_MR.

This study has several important strengths. First, most of the current studies include a follow-up period of 1–2 years after birth; our study was based on a large sample size and a retrospective follow-up period of 5 years. Second, in our study, which is based on a population sample, we examined the associations of various BDs with the U_5_MR and provided probabilities of death for different age periods. There were also several limitations. First, in China, due to economic and information conditions, BDs can be monitored only up to 7 days after birth, but many BDs are still diagnosed after 7 days. So, in our study, we only included BDs diagnosed up to 7 days after birth. Second, child deaths were registered only up to 5 years of age in the maternal and child health system, and mortality calculations could not be extended. We look forward to working with other population death surveillance systems to study the association between BDs and deaths for longer periods and to provide more evidence for BD counseling.

## Data Availability

The data that support the findings of this study are available from the corresponding author upon reasonable request.

## References

[1] WHO. World Health Statistic 2022, (2022)).

[2] He C Liu L Chu Y Perin J Dai L Li X National and subnational all-cause and cause-specific child mortality in China, 1996–2015: a systematic analysis with implications for the sustainable development goals. Lancet Glob Health. (2017) 5:e186–97. 10.1016/S2214-109X(16)30334-5 28007477 PMC5250590

[3] Hug L Alexander M You D Alkema L. Estimation, national, regional, and global levels and trends in neonatal mortality between 1990 and 2017, with scenario-based projections to 2030: a systematic analysis. Lancet Glob Health. (2019) 7:e710–20. 10.1016/S2214-109X(19)30163-9 31097275 PMC6527519

[4] N.H.C.o.t. PRC. China Maternal and Child Health Monitoring Report, (2022).

[5] Benjamin RH Salemi JL Canfield MA Nembhard WN Ganduglia Cazaban C Tsao K Causes of neonatal and postneonatal death among infants with birth defects in Texas. Birth Defects Res. (2021) 113:665–75. 10.1002/bdr2.1879 33586914 PMC8920027

[6] Kurdi AM Majeed-Saidan MA Al Rakaf MS AlHashem AM Botto LD Baaqeel HS Congenital anomalies and associated risk factors in a Saudi population: a cohort study from pregnancy to age 2 years. BMJ Open. (2019) 9:e026351. 10.1136/bmjopen-2018-026351 31492776 PMC6731804

[7] Sattolo ML Arbour L Bilodeau-Bertrand M Lee GE Nelson C Auger N. Association of birth defects with child mortality before age 14 years. JAMA Netw Open. (2022) 5:e226739. 10.1001/jamanetworkopen.2022.6739 35404459 PMC9002336

[8] Singh GK Yu SM. Infant mortality in the United States, 1915–2017: large social inequalities have persisted for over a century. Int J Matern Child Health AIDS. (2019) 8:19–31. 10.21106/ijma.271 PMC6487507 31049261

[9] Downing KF Nembhard WN Rose CE Andrews JG Goudie A Klewer SE Survival from birth until young adulthood among individuals with congenital heart defects: CH STRONG. Circulation. (2023) 148:575–88. 10.1161/CIRCULATIONAHA.123.064400 37401461 PMC10544792

[10] Gili JA Lopez-Camelo JS Nembhard WN Bakker M de Walle HEK Stallings EB Analysis of early neonatal case fatality rate among newborns with congenital hydrocephalus, a 2000–2014 multi-country registry-based study. Birth Defects Res. (2022) 114:631–44. 10.1002/bdr2.2045 35633200 PMC9288486

[11] Bakker MK Kancherla V Canfield MA Bermejo-Sanchez E Cragan JD Dastgiri S Analysis of mortality among neonates and children with spina Bifida: an international registry-based study, 2001–2012. Paediatr Perinat Epidemiol. (2019) 33:436–48. 10.1111/ppe.12589 31637749 PMC6899817

[12] Glinianaia SV Rankin J Tan J Loane M Garne E Cavero-Carbonell C Ten-year survival of children with trisomy 13 or trisomy 18: a multi-registry European cohort study. Arch Dis Child. (2023) 108:461–7. 10.1136/archdischild-2022-325068 36882305

[13] Meyer RE Liu G Gilboa SM Ethen MK Aylsworth AS Powell CM Survival of children with trisomy 13 and trisomy 18: a multi-state population-based study. Am J Med Genet A. (2016) 170A:825–37. 10.1002/ajmg.a.37495 26663415 PMC4898882

[14] Farr SL Riley C Van Zutphen AR Brei TJ Leedom VO Kirby RS Prevention and awareness of birth defects across the lifespan using examples from congenital heart defects and spina bifida. Birth Defects Res. (2022) 114:35–44. 10.1002/bdr2.1972 34921598 PMC8902432

[15] Benjamin RH Nguyen JM Canfield MA Shumate CJ Agopian A. Survival of neonates, infants, and children with birth defects: a population-based study in Texas, 1999–2018. Lancet Reg Health Am. (2023) 27:100617. 10.1016/j.lana.2023.100617 37868647 PMC10589744

[16] Wang Y Liu G Canfield MA Mai CT Gilboa SM Meyer RE Racial/ethnic differences in survival of United States children with birth defects: a population-based study. J Pediatr. (2015) 166:819–26 e1-2. 10.1016/j.jpeds.2014.12.025 25641238 PMC4696483

[17] Coi A Santoro M Pierini A Rankin J Glinianaia SV Tan J Survival of children with rare structural congenital anomalies: a multi-registry cohort study. Orphanet J Rare Dis. (2022) 17:142. 10.1186/s13023-022-02292-y 35351164 PMC8966236

[18] Mauvais-Jarvis F Merz NB Barnes PJ Brinton RD Carrero J-J DeMeo DL Sex and gender: modifiers of health, disease, and medicine. Lancet. (2020) 396:565–82. 10.1016/S0140-6736(20)31561-0 32828189 PMC7440877

[19] Ujagare D Kar A. Birth defect mortality in India 1990–2017: estimates from the global burden of disease data. J Community Genet. (2021) 12:81–90. 10.1007/s12687-020-00487-z 33063164 PMC7846616

[20] Politis MD Bermejo-Sánchez E Canfield MA Contiero P Cragan JD Dastgiri S Prevalence and mortality in children with congenital diaphragmatic hernia: a multicountry study. Ann Epidemiol. (2021) 56:61–69 e3. 10.1016/j.annepidem.2020.11.007 33253899 PMC8009766

[21] Gunne E Lynch SA McGarvey C Hamilton K Lambert DM. Fatal fetal abnormality Irish live-born survival-an observational study. J Community Genet. (2021) 12:643–51. 10.1007/s12687-021-00534-3 34215991 PMC8554881

[22] Murphy SL Kochanek KD Xu J Arias E. Mortality in the United States, 2020. NCHS Data Brief. (2021) (427):1–8. 34978528

[23] Perin J Mulick A Yeung D Villavicencio F Lopez G Strong KL Global, regional, and national causes of under-5 mortality in 2000–19: an updated systematic analysis with implications for the sustainable development goals. Lancet Child Adolesc Health. (2022) 6:106–15. 10.1016/S2352-4642(21)00311-4 34800370 PMC8786667

